# Distributed Least-Squares Estimation of a Remote Chemical Source via Convex Combination in Wireless Sensor Networks

**DOI:** 10.3390/s140711444

**Published:** 2014-06-27

**Authors:** Meng-Li Cao, Qing-Hao Meng, Ming Zeng, Biao Sun, Wei Li, Cheng-Jun Ding

**Affiliations:** 1 Institute of Robotics and Autonomous Systems, Tianjin Key Laboratory of Process Measurement and Control, School of Electrical Engineering and Automation, Tianjin University, No. 92, Weijin Road, Tianjin 300072, China; E-Mails: menglicao@tju.edu.cn (M.-L.C.); qh_meng@tju.edu.cn (Q.-H.M.); sunbiao@tju.edu.cn (B.S.); 2 Department of Computer & Electrical Engineering and Computer Science, California State University, Bakersfield, CA 93311, USA; E-Mail: wli@csub.edu; 3 School of Mechanical Engineering, Hebei University of Technology, Dingzigu Road No.1, Tianjin 300130, China; E-Mail: dcj@hebut.edu.cn

**Keywords:** chemical source localization, wireless sensor networks, distributed estimation, nonlinear least-squares estimation, collaborative in-network processing

## Abstract

This paper investigates the problem of locating a continuous chemical source using the concentration measurements provided by a wireless sensor network (WSN). Such a problem exists in various applications: eliminating explosives or drugs, detecting the leakage of noxious chemicals, *etc.* The limited power and bandwidth of WSNs have motivated collaborative in-network processing which is the focus of this paper. We propose a novel distributed least-squares estimation (DLSE) method to solve the chemical source localization (CSL) problem using a WSN. The DLSE method is realized by iteratively conducting convex combination of the locally estimated chemical source locations in a distributed manner. Performance assessments of our method are conducted using both simulations and real experiments. In the experiments, we propose a fitting method to identify both the release rate and the eddy diffusivity. The results show that the proposed DLSE method can overcome the negative interference of local minima and saddle points of the objective function, which would hinder the convergence of local search methods, especially in the case of locating a remote chemical source.

## 1. Introduction

At present, advances in chemical sensing technology [1] have made it applicable to maintain a wireless network of inexpensive and reliable chemical sensors for environment monitoring. An important application of such a WSN is CSL, which consists of not only measuring the concentration of the objective chemical substance but also locating the chemical source using the concentration measurements. Designing an efficient CSL method may greatly reduce both human injuries and financial losses [2]. Compared with mobile sensors, a static WSN can cover a large area at a faster speed. This advantage is more remarkable when the target area constrains the mobility of the mobile sensors. For example, in the case of locating the source of a smoke cloud at the initial stage of a potential conflagration in a mountain, a WSN can be rapidly self-organized by a large number of sensor nodes which are deployed from a plane flying over the cloud, while the way of existing mobile sensors would be hindered due to the complicated obstacles.

However, the working time of WSN would be limited if heavy communication burdens are imposed, since the sensor nodes are mainly powered up by batteries which cannot be easily charged on site. This problem makes the power consumption a key factor during the design of applications based on WSNs. To save the limited power, the most commonly used method is to reduce the power consumed by wireless communication. Specifically, this target can be realized by utilizing distributed methods which avoid transmitting raw measurements to the sink node. Moreover, the robustness of the distributed method is better than the centralized one, *i.e.*, for distributed methods, any single node having problem does not influence the whole result.

The problem of CSL using a network of chemical sensors has been intensively investigated [3–10] since the seminal work of Nehorai *et al.* [11]. Among these works, least-squares estimation (LSE) based methods require no statistical assumptions about the measurement errors, since the estimate is chosen to provide a “best” fit to the observed measurements in a deterministic sense [12,13]. Nevertheless, the objective function of LSE based CSL using WSN has multiple local optima and saddle points. Thus, the traditionally used search techniques may converge locally to a suboptimal solution. Matthes *et al.* [4] proposed a superior unimodal objective function which can largely reduce the difficulty of searching the single maximum located at the real source location. However, the raw concentration measurements should be gathered together in order to calculate the objective function. This process of gathering the raw measurements at the sink node conflicts with the scheme of distributed methods.

This paper proposes a distributed, global convergent LSE method for solving the problem of CSL using WSNs. The problem of minimizing the objective function of LSE based CSL using WSNs, *i.e.*, the sum of squared measurement errors, is decomposed into multiple sub-problems of minimizing individual summands, each of which is associated with the information of an individual sensor node and thus can be solved locally on the associated sensor node. Aiming to minimize an individual summand, we derive a set of probable source locations by substituting the noisy measurement to the concentration distribution model. An estimate of the source location is locally determined from each of the location sets by empirically maximizing the probability of being the real source location based on the prior information about the source location. Then, the problem of CSL using a WSN can be solved by comprehensively incorporating the information of these local estimates into a global estimate. We consider a convex combination of these local estimates as the global estimate of the source location. The convex combination of these local estimates, which are distributed on individual sensor nodes, is realized in a distributed manner using the distributed average consensus algorithm proposed in [14]. Finally, the above-mentioned process repeats iteratively until the termination condition is satisfied and the global estimate is considered as the prior information about the source location for determining the local estimates at the next iteration. The proposed method was assessed in both simulations and real experiments, including the case of locating a remote chemical source.

The rest of this paper is organized as follows: the chemical concentration distribution model, the sensor-source distance function, and the measurement model are described in Section 2. The DLSE method for CSL using WSN is proposed in Section 3. The results of numerical simulations and real experiments are presented in Sections 4 and 5, respectively. Some concluding remarks are given in Section 6.

## 2. Formulation and Preliminaries

In this section, the advection-diffusion chemical concentration distribution model is introduced, and then the distance between the unknown source and the sensor node is represented as a function of their relative angle and the theoretical concentration at the sensor node location. Finally, the concentration measurement model of the sensor nodes in the WSN is presented.

### 2.1. Chemical Concentration Distribution Model

**Figure 1.**
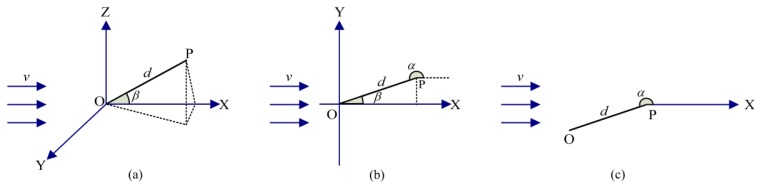
Coordinate systems used in this paper. (**a**) 3-dimensional Cartesian coordinate system; the distance between *O* and *P* is *d* cm, the angle between *OP* and *OX* is *β*. (**b**) 2-dimensional Cartesian coordinate system; the case that *P* is located on the X*O*Y plane, and *α* is the angle from the positive *x*-axis to *PO*. (**c**) the polar coordinate system in which *P* is taken as the origin, so that the position of *O* can be determined based on the position of *P*.

The advection-diffusion model proposed in [15] is adopted in this paper. In the theory of eddy diffusion in the atmosphere, the diffusion rate at a certain point under each pressure level is parameterized by the eddy diffusivity, *K* cm^2^/s. The concentration of the chemical substance, *c* g/mL, can reach a steady state in a homogeneous wind field in which the eddy diffusivity is the same at every point [15]. As shown in Figure 1, the continuous chemical source located at point *O* is releasing at a rate, *q* g/s. The released chemical substance is advected by a homogeneous wind [16], the speed of which is *v* cm/s along the positive *x*-axis. The sensor, which is represented by point *P* at (*x*,*y*,*z*), lies in the plume caused by the chemical source. The steady concentration at the point (*x*,*y*,*z*) satisfies:

(1) $$v\frac{\partial c}{\partial x}=K\nabla^{2}c$$

The concentration distribution is symmetry about the *x*-axis due to the homogenous flow, and then Equation (1) becomes:

(2) $$\left(v\cos\beta \frac{\partial }{\partial d}-\frac{v\sin\beta }{d}\frac{\partial }{\partial \beta }\right)c=\frac{K}{d^{2}}\left[\frac{\partial }{\partial d}\left(d^{2}\frac{\partial c}{\partial d}\right)+\frac{1}{\sin\beta }\frac{\partial }{\partial \beta }\left(\sin\beta \frac{\partial c}{\partial \beta }\right)\right]$$

where *β* is the angle between *OP* and *OX* [15].

Considering that the concentrations at the source location and the point extremely far away from the source (*i.e.*, *d* → +∞) approximately equal +∞ and zero, respectively. Then, the solution of Equation (2) is:

(3) $$c\left(x,y,z\right)=\frac{q}{4\pi Kd}\exp\left[-\frac{vd\left(1-\cos\beta \right)}{2K}\right]$$

In the case studied here, the chemical source and the sensor nodes are situated on an impenetrable floor. To analyze the concentration distribution on the floor, we first suppose that the chemical source is placed at (0,0,*h*). Since no chemical matters can diffuse through the impenetrable floor, the concentration at *P* can be calculated using the method of mirror images [17] as follows:

(4) $$c\left(x,y,z\right)=\frac{q}{4\pi Kd^{\prime }}\exp\left[-\frac{vd^{\prime }\left(1-\cos\beta \right)}{2K}\right]+\frac{q}{4\pi Kd^{\prime \prime }}\exp\left[-\frac{vd^{\prime \prime }\left(1-\cos\beta \right)}{2K}\right]$$

where $d^{\prime }=\sqrt{x^{2}+y^{2}+{\left(z-h\right)}^{2}}$ and $d^{\prime \prime }=\sqrt{x^{2}+y^{2}+{\left(z+h\right)}^{2}}$. Then, substituting *h* = 0 and *z* = 0 into Equation (4) for the concentration in our case, the concentration at *P* is given by:

(5) $$c\left(x,y\right)=\frac{q}{2\pi Kd}\exp\left[-\frac{vd\left(1-\cos\beta \right)}{2K}\right]$$

More generally, when the chemical source is located at another point (*x*_0_,*y*_0_) in the 2-dimensional coordinate system, the associated distance will be $d=\sqrt{{\left(x-x_{0}\right)}^{2}+{\left(y-y_{0}\right)}^{2}}.$

To emphasize the importance of the node locations in determining the source location, the location of the chemical source is transferred into the polar coordinate system in which the sensor location *P* is taken as the origin, as shown in Figure 1c. Then, the coordinate (*x*_0_,*y*_0_) is expressed as:

(6) $$\{\begin{array}{cc} x_{0} & =x+d\cos\alpha \\ y_{0} & =y+d\sin\alpha \end{array}$$

where *α* ∈ (0, 2*π*] is the angle from the positive *x*-axis to the broken line pointing from the measuring location to the source. The relationship between *α* and *β* is shown in Figure 1b. Obviously, *α* = *β*+*π* and Equation (5) becomes:

(7) $$\begin{array}{ll} c\left(x,y\right) & =\frac{q}{2\pi Kd}\exp\left\{-\frac{vd\left[1-\cos\left(\alpha -\pi \right)\right]}{2K}\right\}\\ & =\frac{q}{2\pi Kd}\exp\left\{-\frac{vd\left(1+\cos\alpha \right)}{2K}\right\} \end{array}$$

The advection-diffusion model introduced above was also referred to as Robert's model in [18]. Some of other studies based on this model can be found in [4,17,19,20].

### 2.2. Deriving the Sensor-Source Distance Function

Based on the concentration distribution model in Equation (7), given the concentration *c*, flow speed *v* and the parameters *K* and *q*, the distance between the chemical source and the sensor node can be expressed as a function of *α* by reverse derivation.

First, applying the equation $\cos\alpha =2\cos^{2}\frac{\alpha }{2}-2\sin^{2}\frac{\alpha }{2}$ to Equation (7), we get

(8) $$c=\frac{q}{2\pi K}/\left[d\cdot \exp\left(\frac{vd\cos^{2}\frac{\alpha }{2}}{K}\right)\right]$$

Then, moving the denominator in the right-hand-side of Equation (8) to the left-hand side and multiplying both sides with $\frac{v\cos^{2}\left(\overset{\alpha }{\;}/\underset{2}{\;}\right)}{Kc}$, we obtain

(9) $$\frac{vd\cos^{2}\frac{\alpha }{2}}{K}\exp\left(\frac{vd\cos^{2}\frac{\alpha }{2}}{K}\right)=\frac{vq\cos^{2}\frac{\alpha }{2}}{2\pi K^{2}c}$$

Based on the principle branch of the Lambert W function, *i.e.*, *W*_0_(*s*), *s* > 0, which satisfies *W*_0_(*s*) exp[*W*_0_(*s*)] =*s*, *s* > 0 [21], when *α* ≠ *π*, Equation (9) can be rewritten as:

(10) $$\frac{vd\cos^{2}\frac{\alpha }{2}}{K}=W_{0}\left(\frac{vq\cos^{2}\frac{\alpha }{2}}{2\pi K^{2}c}\right)$$

Finally, the sensor-source distance *d* can be represented as follows:

(11) $$d=\{\begin{array}{cc} \frac{q}{2\pi Kc}, & \alpha =\pi \\ \frac{K}{v\cos^{2}\frac{\alpha }{2}}W_{0}\left(\frac{vq\cos^{2}\frac{\alpha }{2}}{2\pi K^{2}c}\right), & \text{others} \end{array}$$

Since the principle branch of the Lambert W function is single-valued and monotonically increasing, according to Equation (11), *d* is inversely proportional to *c* when other variables are kept constant.

### 2.3. Measurement Model

Suppose there is a WSN that consists of *n* chemical sensor nodes randomly deployed at known global coordinates ***x****_i_* = (*x_i_*,*y_i_*),*i* ∈ [1,*n*] on the same 2-dimensional field as the chemical source deployed at ***x****_0_* = (*x_0_*,*y_0_*). Note that the boldface lowercase letter denotes the coordinate vector of a sensor node, as well as the sensor node itself, which is easy to be distinguished in the rest of this paper. After the steady state of concentration distribution is reached, the measurements {*z_i_*, *i* ∈ [1,*n*]} at the same sampling time can be formulated as:

(12) $$z_{i}=c_{i}+e_{i}$$

where *c_i_* represents the theoretical concentration at the location of ***x**_i_*, *e_i_* stands for the measurement error which is mainly comprised of the errors caused by the uncontrollable drift of the sensors and the sensors' response to foreign substances due to the poor selectivity of the commonly used chemical sensors [2]. Typically, it can be considered that the error terms for all the sensor nodes at the same sampling time satisfies the normal distribution with a positive mean value [11]. Due to the relatively small variance of the normal distribution of *e_i_*,*i* ∈ [1, *n*], the value of *z_i_*,*i* ∈ [1, *n*] can roughly represent its signal-to-noise ratio (SNR), *i.e.*, |(*z_i_*/*e_i_*) − 1| [6,7]. For a better localization performance, a measurement threshold is set to eliminate the measurements with comparatively low SNRs. The nodes with measurements that are smaller than the threshold *z_th_* will be scheduled to hibernate, and the remaining *l* (*l* < *n*) nodes will participate in the localization.

## 3. Distributed Least-Squares Estimation Method for CSL Using WSN

**Figure 2.**
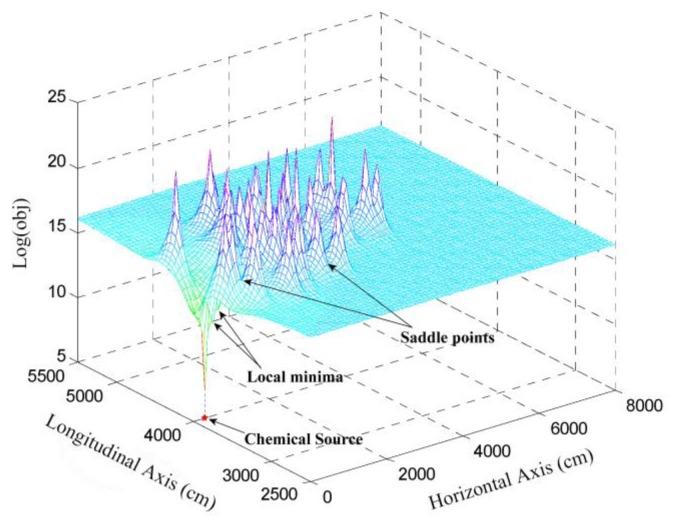
Mesh graph of the objective function in Equation (13); the actual source is located at (3,40) m. The unit of the objective function is ppm^2^. Some of the local minima and the saddle points are pointed out. More details about the simulation setup are given in Section 4.

In the LSE-based CSL using a WSN, the objective function to be minimized is the sum of squares of the *l* participating scattered sensor nodes' measurement errors [22], which is as follows:

(13) $$\text{obj}\left(x_{0}\right)=\sum_{i=1}^{l}{\left[z_{i}-c_{i}\left(x_{0}\right)\right]}^{2},\;x_{0}\in {ℜ}^{2}$$

where *z_i_*, *i* ∈ [1,*l*] and *c_i_*(***x***_0_), *i* ∈ [1,*l*] are known measurements and unknown theoretical concentration (since ***x***_0_ is unknown during the localization), respectively. By varying the location of ***x***_0_ in its domain, *i.e.*, ***x***_0_ ∈ ℜ^2^, the distribution of this objective function can be obtained. The logarithm of this distribution is illustrated in Figure 2. There is a global minimum located at the actual source location, while there are some local minima near the global minimum, as well as some saddle points around the local maxima.

As shown in Figure 2, there is a global minimum located at the actual source location. It is straightforward that the objective function in Equation (13) would achieve its global minimum, when the *l* summands, *i.e.*, [*z_i_* − *c_i_* (***x***_0_)]^2^, reach their minima simultaneously. The fact that [*z_i_* − *c_i_* (***x***_0_)]^2^ is irrelevant to all the sensor nodes except ***x****_i_*, motivates the idea of designing a local estimation of the source location, which is aiming to minimize [*z_i_* − *c_i_* (***x***_0_)]^2^, for ***x****_i_*.

**Figure 3.**
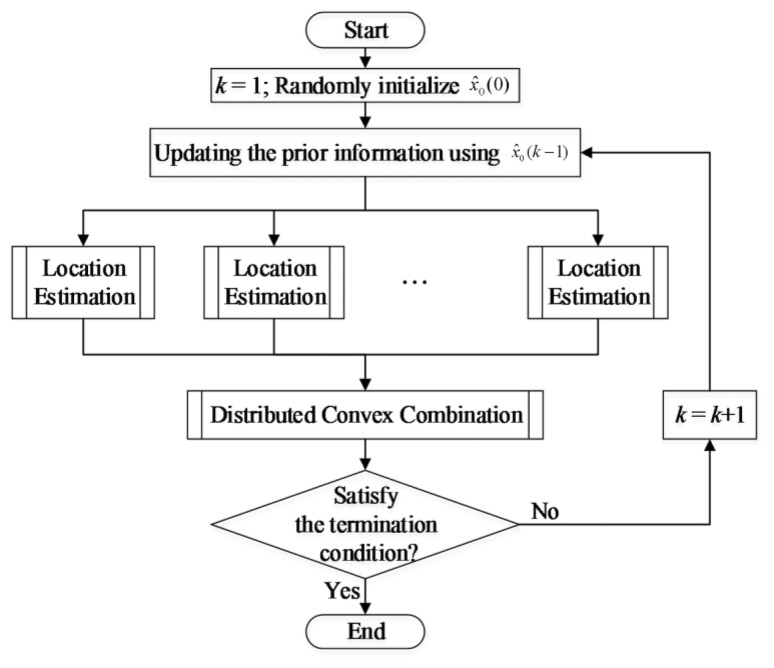
The flow chart of the proposed localization method. The location estimations are conducted locally on all of the sensor nodes which have measured above-threshold concentration values. ***x̂***_0_(*k*) is the global estimate at the *k*-th iteration of localization.

After the estimates of these local estimations are achieved, they are convexly combined to form the global estimate by using an average consensus algorithm. The overall scheme of the proposed method is given in Figure 3, in which the sub-processes are detailed in the following subsections.

### 3.1. Local Estimation of the Source Location

In Section 2, the location of the chemical source has been transformed into the polar coordinate system, in which the location of sensor node is taken as the origin. According to Equation (11), when *α* ≠ *π* the coordinates of the source location can be further expressed as functions of *α* as follows:

(14) $$\{\begin{array}{c} x_{0}=x+\frac{K(1-\tan^{2}\frac{\alpha }{2})}{v}W_{0}\left(\frac{vq\cos^{2}\frac{\alpha }{2}}{2\pi K^{2}c}\right)\\ y_{0}=y+\frac{2K}{v}\tan\frac{\alpha }{2}W_{0}\left(\frac{vq\cos^{2}\frac{\alpha }{2}}{2\pi K^{2}c}\right) \end{array},\alpha \neq \pi$$

For the case that *α* ≠ *π*, the source location is $\left(x_{0},y_{0}\right)=(x-\frac{q}{2\pi Kc},y)$. However, the calculation of Equation (14) involves determining the theoretical concentration *c*, which is impossible to be obtained due to the unknown source location. Since *e_i_*, *i* ∈ [1,*l*] are minor compared with *c_i_*, *i* ∈ [1,*l*], the measurement *z_i_*, *i* ∈ [1,*l*] can be considered as an approximate estimate of *c_i_*, *i* ∈ [1,*l*]. Then, by substituting *z_i_* for *c* in Equation (14) and changing *α* in its domain (0, 2*π*], we can determine a set of points which are approximate estimates of the source location. The point set corresponding to *z_i_*, *i* ∈ [1,*l*] is denoted by $S_{i}=\left\{x_{0}^{i}\right\},i\in \left[1,l\right]$. In the context of probabilistic inference, the points in $S_{i}=\left\{x_{0}^{i}\right\},i\in \left[1,l\right]$ can be considered as the probable source locations inferred based on *z_i_*, *i* ∈ [1,*l*]. Compared with the domain of ***x***_0_, *i.e.*, ***x***_0_ ∈ ℜ^2^, the set $S_{i}=\left\{x_{0}^{i}\right\},i\in \left[1,l\right]$ is substantially refined, and thus is referred to as the refined sample space (*i.e.*, the set of all possible results) for the subsequent local estimation on ***x****_i_*, *i* ∈ [1,*l*].

**Figure 4.**
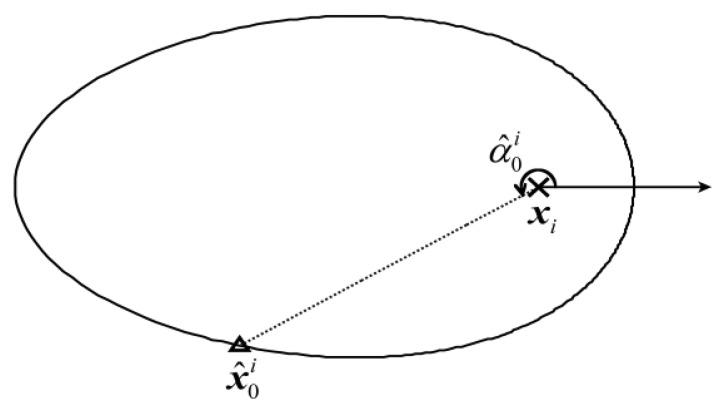
The oval formed by all the points in a single refined sample space, *i.e.*, *S_i_*, *i* ∈ [1,*l*]. The cross and the triangle denotes the location of ***x****_i_*, *i* ∈ [1,*l*] and the local estimate, *i.e.*, ${\hat{x}}_{0}^{i},i\in [1,l]$, respectively. The arrow is the polar axis, the direction of which is also the flow direction.

After *z_i_* is substituted for *c*, Equation (14) becomes an equation set with a single unknown *α* whose domain is (0, 2*π*]. Therefore, as shown in Figure 4, the problem of estimating ***x***_0_ can be transformed to another problem of estimating the angle of the vector pointing from ***x****_i_* to ***x***_0_, *i.e.*, $\alpha_{0}^{i}$. Although it is difficult to assign a rigorous theoretical probability of being the actual source location to each point in *S_i_*, *i* ∈ [1,*l*], if a predictive location of the source exists, it can be empirically considered that the point with the same polar angle as the predictive location has the largest probability of being the actual source location among all the points in *S_i_*, *i* ∈ [1,*l*]. Inspired by the process of iterative estimation, the global estimate ***x̂***(*k*−1) is considered as the prior information (predictive location of the source) for the local estimations at the *k*-th iteration of localization. Let us define ***α̂***(*k*) as the angle between the vector ***x̂***(*k*−1)−***x**_i_* and the positive direction of the polar axis. Then, a local estimate of the source location can be considered as:

(15) $$\begin{array}{l} {\hat{x}}_{0}^{i}\left(k\right)=x_{0}^{i}\left(z_{i}\left(k\right),{\hat{\alpha }}_{i}\left(k\right)\right),i\in \left[1,l\right]\\ {\hat{\alpha }}_{i}\left(k\right)≜\text{angle}\left({\hat{x}}_{0}\left(k-1\right)-x_{i}\right) \end{array}$$

where $x_{0}^{i}\left(z_{i}\left(k\right),{\hat{\alpha }}_{i}\left(k\right)\right)$ denotes an estimate of the source location determined by *α̂_i_*(*k*) from the refined sample space $S_{i}=\left\{x_{0}^{i}\right\},i\in \left[1,l\right]$ calculated using *z_i_* (*k*). This process of local estimation can be considered as a quasi-maximum a posterior estimation because the maximum posterior probability is determined empirically, rather than theoretically, based on the location of ***x̂***_0_(*k*−1). Note that, at the beginning of the localization there is no prior information about the source location. To start the localization, an initial point must be set as the prior information for the first iteration of localization. Since the initial point has little influence on the localization performance (see Section 4.3), it can be randomly initialized.

### 3.2. Combining the Local Estimates to Form the Global Estimate

Section 3.1 presents a framework for the local estimation of the chemical source location based on the prior information about the predictive location of the source. However, it does not detail how this prior information is achieved and how it is accessible to the participating scattered sensor nodes. Moreover, the individual local estimates can only minimize an individual summand of the objective function in Equation (13) with the maximum probability. Thus, to comprehensively consider all the summands of the objective function in Equation (13), these local estimates are combined to form a global estimate, which serves as the prior information about the source location at the next iteration of localization.

#### 3.2.1. Convex Combination of the Local Estimates

A convex combination of the local estimates at the *k*-th iteration of localization, *i.e.*, ${\hat{x}}_{0}^{i}\left(k\right)$, is considered as the *k*-th global estimate ***x̂***_0_(*k*):

(16) $${\hat{x}}_{0}\left(k\right)=\sum_{i=1}^{l}w_{i}\left(k\right){\hat{x}}_{0}^{i}\left(k\right)$$

where *w_i_* (*k*) satisfies $\overset{l}{\underset{i=1}{\text{∑}}}w_{i}\left(k\right)=1$ and *w_i_* (*k*) > 0 (rendering the combination in Equation (16) as convex), ${\hat{x}}_{0}^{i}\left(k\right)$ is calculated using Equation (15).

**Figure 5.**
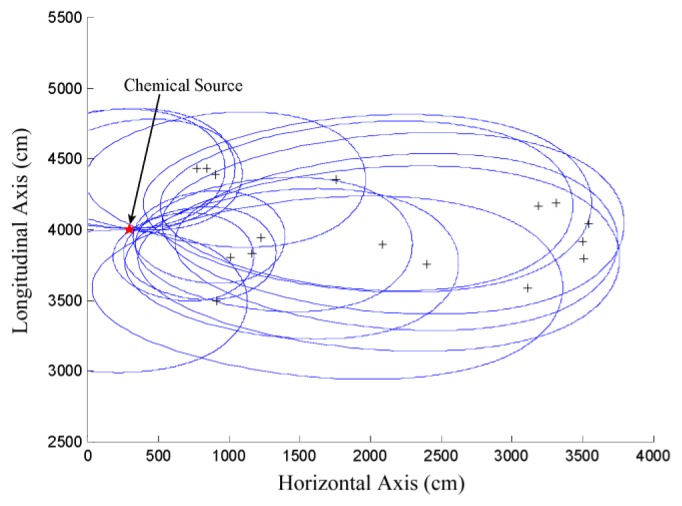
The positional relationship between the actual chemical source location and the points in different refined sample spaces, *i.e.*, *S_i_*, *i* ∈ [1,*l*], which are represented by different ovals. The sensor nodes are denoted as crosses. The simulation setup used to generate this figure is the same as that used in generating Figure 2, which will be detailed in Section 4.

To set reasonable weights for the local estimates in Equation (16), let us consider the positional relationship between ***x***_0_ and the points in *S_i_*, *i* ∈ [1,*l*] from which the local estimates are selected. When the different contaminated measurements *z_i_*, *i* ∈ [1,*l*] are substituted for *c* in Equation (14), the size of the associated ovals formed by the points in different refined sample spaces (*i.e.*, *S_i_*, *i* ∈ [1,*l*]) are different. The oval generated by substituting a larger *z_i_* for *c* in Equation (14) is smaller than that generated using a smaller *z_i_*. This is mainly because *d* will decrease if *c* increases and other parameters are kept constant according to Equation (11). Moreover, as shown in Figure 5, the points on a smaller oval are generally closer to ***x***_0_ than those on a bigger oval. Therefore, it is reasonable to construct a direct proportion between *w_i_* (*k*) and *z_i_* (*k*) to make the sequence of ***x̂***_0_(*k*) approach ***x***_0_. The weights *w_i_* (*k*) are tentatively set as the proportion of *z_i_* (*k*) in the sum of all the *l* measurements, as in Equation (17), and achieve good performance in the results presented in Sections 4 and 5:

(17) $$w_{i}\left(k\right)=\frac{z_{i}\left(k\right)}{\overset{l}{\underset{j=1}{\text{∑}}}z_{j}\left(k\right)},i\in \left[1,l\right]$$

#### 3.2.2. Distributed Average Consensus Algorithm

It is readily seen that there are two individual accumulating operators in Equation (16) and Equation (17). Moreover, the accumulating results, *i.e.*, ***x̂***_0_(*k*) and the denominator in Equation (17), should be accessible to all the participating scattered sensor nodes. The centralized processing method in which a fusion center gathers the summands from and then transmits the sum back to all the sensor nodes is not power-efficient for WSN, and thus is avoided in this paper. Since the sum can be considered as the product of the amount and the average of the summands, the process of accumulation can be transformed to an averaging process and an additional multiplication between the amount and the associated average as follows:

(18) $$\overset{l}{\underset{i=1}{\text{∑}}}f_{i}\left(0\right)=l\times \text{Avg}\left\{f_{i}\left(0\right),i\in \left[1,l\right]\right\}$$

where *Avg*{·} means the averaging operator, and *f_i_*_0_, *i* ∈ [1,*l*] are the initial states of the values to be averaged. For example, in the case of calculating Equation (16), $f_{i}\left(0\right)=w_{i}(k){\hat{x}}_{0}^{i}\left(k\right),i\in \left[1,l\right]$. Then, the distributed average consensus algorithm in [14] can be used to calculate the averaging term in Equation (18), which is involved in the convex combination of local estimates twice, *i.e.*, in calculating Equations (16) and (17).

**Figure 6.**
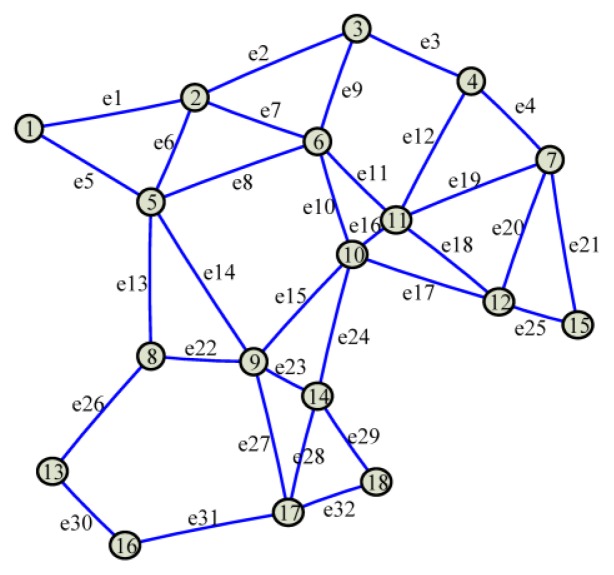
The associated graph of the mesh architecture in Figure 1d. The sensor nodes are denoted as circles, in which the numbers are the indexes of the associated nodes. Communication links within the neighboring ranges are represented by solid lines, and the indexes of these edges are given aside them.

The distributed average consensus algorithm means that only local communications within the neighboring set of each participating scattered sensor node are needed during the averaging procedure [23]. The steps of adopting this algorithm to our specific problem are elaborated as follows:

Step 1 *Generate the corresponding graph and the Laplacian matrix*. Suppose each participating scattered sensor node can only communicate with the sensor nodes within its neighboring range. A graph describing the corresponding network architecture is generated. In the generated graph, there is an edge between two nodes which can communicate with each other. Then, the Laplacian matrix (denoted by *L*) of the graph is calculated as *L* = *AA^T^*, where *A* is the incidence matrix of the graph. Since *A* is a matrix with *l* rows and *g* columns, where *g* is the number of edges in the graph, *L* is a *l*-dimensional square matrix.
  For example, as shown in Figure 6, there are *l* = 18 vertexes and *g* = 32 edges in the graph corresponding to the mesh architecture in Figure 1d, so the corresponding Laplacian matrix is a 18-dimensional square matrix.
Step 2 *Calculating the modulus matrix*. A modulus matrix, which is denoted by *M*, is calculated as follows:
  (19) $$M=I-\frac{2}{\lambda_{1}\left(L\right)+\lambda_{l-1}\left(L\right)}\cdot L$$
  where *λ_i_* (*L*), *i* ∈ [1,*l*] is the *i*-th largest eigenvalue of *L*, and *I* is the identity matrix. Then, the nonzero elements in the *i*-th row of *M*, which is denoted by *M_i_*, are transmitted to *x_i_*. These nonzero elements corresponds to ***x****_i_* and its neighboring nodes. For example, in Figure 5, there are three nonzero elements in *M*_1_, *i.e.*, *M*_11_, *M*_12_ and *M*_15_, which correspond to ***x***_1_, ***x***_2_ and ***x***_5_, respectively. Since only a few nodes are connected with each participating scattered sensor node, the matrix *M* is sparse and the number of the elements to be transmitted is small.
Step 3 *Performing the iteration of averaging.* Since the proposed localization algorithm and the average consensus algorithm used here are both iterative algorithms, it is important to distinguish them from each other. The iteration in the former is called the iteration of localization (counted by *k*), while the iteration in the latter is called the iteration of averaging (counted by *t*). In the *k*-th iteration of localization, two whole processes of iterative averaging (*i.e.*, from the initial state to the convergent state) should be conducted to calculate $\overset{l}{\underset{j=1}{\text{∑}}}z_{j}\left(k\right)$ in Equation (16) and $\overset{l}{\underset{i=1}{\text{∑}}}w_{i}\left(k\right){\hat{x}}_{0}^{i}\left(k\right)$ in Equation (17). In the *t*-th iteration of averaging, ***x****_i_*, *i* ∈ [1,*l*] first transmits its own value *f_i_*(*t*), *i* ∈ [1,*l*], t ≥ 1 (an updating version of *f_i_*(0),*i* ∈ [1,*l*]) to its neighboring nodes, and then updates it by adding up the products of the nonzero elements and the values received from its neighbors. For example, according to the topology illustrated in Figure 6, *f*_1_(*t*) = *M*_11_*f*_1_(*t* − 1) + *M*_12_*f*_2_(*t* − 1) + *M*_15_*f*_5_(*t* − 1) at the *t*-th iteration of averaging. This step actually realizes a matrix multiplication as follows:
  (20) $$\vec{f}\left(t+1\right)=M\times \vec{f}\left(t\right)$$
  where *f⃗*(*t*) is a column vector that consists of *f_i_*(*t*), *i* ∈ [1,*l*]. Note that Equation (20) coincidentally involves *l* local convex combinations on the *l* participating sensor nodes, which can be distinguished from the global convex combination in Equation (16) by analyzing their different summands. The fast convergence of Equation (20), which means *f_i_*(*t*), *i* ∈ [1,*l*] would converge to $\left({\text{∑}}_{i=1}^{l}f_{i}\left(0\right)\right)/l$ in a few iterations of consensus, has been proved in [14].

If the variation of the global local estimation, *i.e.*, Δ***x̂***_0_(*k*)=***x̂***_0_(*k*)−***x̂***_0_(*k*−1), has not exceeded a predefined range threshold $\Delta {\hat{x}}_{0}^{th}$ for three iterations of localization, the process of localization will be terminated. The sink node should know the indexes and locations of ***x****_i_*, *i* ∈[1,*l*] for generating the corresponding graph, and ***x****_i_*, *i* ∈[1,*l*] should know *l* and the nonzero elements in *M_i_*,*i* ∈[1,*l*] for performing the iterations of consensus. Thus, some information exchanges between the sink and the participating scattered sensor nodes are engaged in the first two steps. However, the first two steps are conducted only once at the first iteration of localization, *i.e.*, *k* = 1. Therefore, the communication burdens in these two steps are acceptable. Basically, there should be some routine communications between the sink and the scattered nodes to guarantee the conventional operations of the WSN.

## 4. Simulation Results

In this section, the localization performance of the proposed DLSE method is assessed through simulations. First, the basic simulation setup, which is used to generate Figures 2 and 5 and served as the prototype of the following simulations, is described. Then, the process of distributed averaging, which is the premise of distributed implementation of DLSE, is demonstrated. Afterwards, the localization performance of DLSE is compared with the trust-region-reflective algorithm which is recommended for solving centralized nonlinear LSE problem. Finally, the ability of locating a remote chemical source using DLSE is assessed.

### 4.1. Basic Simulation Setup

The simulated WSN was composed of 300 nodes that were randomly distributed over an 80 m × 80 m square. The lower left corner and bottom margin of this square were taken as the origin and abscissa axis of the global Cartesian coordinate system, respectively. In Figures 2 and 5, the chemical source was located at (300,4000) cm, and the chemical substances released from the source was diffused by the flow with a near-surface velocity of *v* = 70 cm/s. The eddy diffusivity, *K*, was set as 10^4^ cm^2^/s. The unit of the release rate in Robert's original paper [15] is g/s, and thus the associated unit of concentration is g/cm^3^. At 20 °C and standard atmospheric pressure, the mass concentration in g/cm^3^ can be transferred to the volume concentration in the most commonly used unit, ppm, by multiplying an exponential (22.4/*M*) × 10^9^, where *M* is the molecular weight of the released substance. The release rate *q* in our simulations was set as 114 g/s, which can cause the concentration range up to dozens of ppm when *M* = 46 (*i.e*., the molecular weight of ethanol) and the sensor-source distance is tens of meters. Referring to [7], the mean and standard deviation of the measurement errors were 10^−5^ kg/m^3^ and 8 × 10^−6^ kg/m^3^. Correspondingly, the concentration threshold was set as 10^−5^ kg/m^3^ to eliminate the measurements with extremely low SNRs.

By setting a presumed chemical source at the center of each of the mesh grids on the node-deployment square, which is denoted as $x_{0}^{\prime }$, a group of $c_{i}^{\prime },i\in \left[1,l\right]$ can be calculated by substituting $x_{0}^{\prime }$ for ***x***_0_ in Equations (6) and (7). Then, the value of the objective function with respect to different presumed source locations can be calculated by substituting different groups of *z_i_*, *i* ∈[1,*l*] and the corresponding $c_{i}^{\prime },i\in \left[1,l\right]$ to Equation (13). The logarithmic transform of these values are illustrated in Figure 2. According to Figure 2, a global minimum appears near the actual source location, while there are also several local minima and saddle points at other locations in the square. To generate Figure 5, the measurements of 30 participating scattered sensor nodes, *i.e.*, *z_i_*, *i* ∈[1, 30] were substituted for *c* in Equation (14).

### 4.2. Demonstrating the Process of Distributed Averaging

As mentioned in Section 3, the convex combination of local estimates is calculated by using a distributed average consensus algorithm, which serves as the prerequisite of the distributed implementation of our method. Thus, before assessing the localization performance of our method, it is necessary to demonstrate the process of distributed averaging.

**Figure 7.**
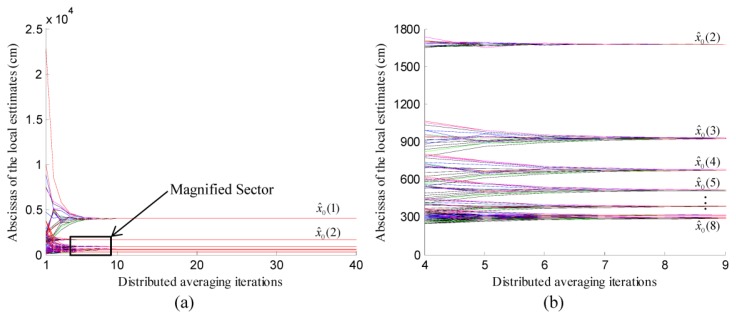
The variation of the abscissas of local estimates in eight successive iterations of localization. To get a clear illustration, the sector surrounded by the black rectangle in (**a**) is magnified and displayed in (**b**).

At the *k*-th iteration of localization, the coordinates of the local estimates ***x̂***_0_(*k*), *i*∈[1,*l*] were convexly combined to form the global estimate, *i.e.*, ***x̂***_0_, which were considered as the initial values, *i.e.*, *f*_*i*0_=***x̂***_0_(*k*), *i*∈[1,*l*]. Then, these values were updated by using the values from the neighboring nodes at each iteration of averaging. The abscissas of the local estimations, *i.e.*, ***x̂***_0_(*k*), *i*∈[1,*l*], at different iterations of localization were illustrated in Figure 7. As shown in Figure 7, the distributed averaging of ***x̂***_0_(*k*), *i*∈[1,*l*] was performed eight times, which were associated with eight iterations of localization. At each time of the averaging, the abscissas maintained by different participating scattered sensor nodes converged to the abscissa of the global estimate, *i.e.*, ***x̂***_0_(*k*), *k*∈[1,8], in about ten iterations of consensus. Moreover, the abscissas of these global estimates converged near the abscissa of the actual chemical source which was located at (300,4000) cm.

### 4.3. The Influence of the Initial Point

The trust-region-reflective (TRR) algorithm [24], which is recommended in Matlab, was used to solve the standard centralized nonlinear LSE of the source location and as the benchmark to assess the performance of our method. The TRR algorithm, which is based on the interior-reflective Newton method, is a subspace trust-region method. Trust region approaches approximate the function to be minimized with a simpler function, which can reasonably reflect the behavior of the original function in a neighborhood around the current point, *i.e.*, the trust region. Then, the trust-region sub-problem, which minimizes the simpler substituted function over the trust region, is solved at each iteration of trust region approaches. The trust-region methods are local search methods since the solution can only move in the trust regions. Thus, TRR is a typical local search algorithm, although the structure of the nonlinear LSE problem is exploited in TRR to enhance efficiency.

**Figure 8.**
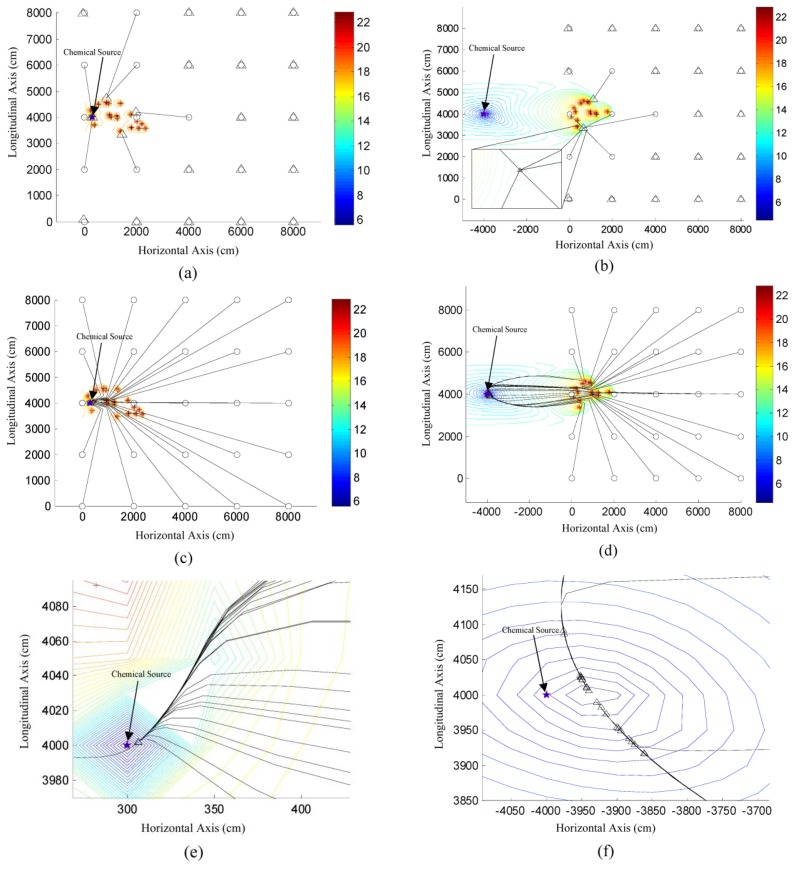
The influence of initial point on the localization performance of TRR and DLSE. The initial points, sensor nodes and the final global estimates are denoted as circles, crosses and triangles, respectively. The source is located at (300,4000) cm in (a) and (c), while it is located at (−4000,4000) cm in (b) and (d). (**a**) and (**b**) show the localization results of TRR, while (**c**) and (**d**) illustrate the variation of global estimates in the process of performing DLSE. (**e**) and (**f**) are the magnified versions of a small rectangular area around the source in (c) and (d), respectively.

The influence of initial point on the localization performance of TRR and DLSE were assessed in two different cases, and the results are illustrated in Figure 8. Note that in Figure 8, the initial points are directly connected to the associated final estimates for TRR, while the intermittent global estimates in a single localization were connected to each other for DLSE. As shown in Figure 8, TRR succeeded several times when the initial point was near the source location, otherwise, it would be stagnated at some of the local optima or failed in starting the search. Contrarily, DLSE succeeded from all of the initial points in the two cases, and achieved relatively small localization errors compared to the large size of the node deployment area. Therefore, the locations of initial points hardly influence the localization performance of DLSE. In other words, DLSE can overcome the problem of poor convergence caused by the multiple local minima and saddle points of the objective function in Equation (13), which would obstruct the convergence of TRR and other local search algorithms.

### 4.4. The Performance of Locating a Remote Chemical Source

**Figure 9.**
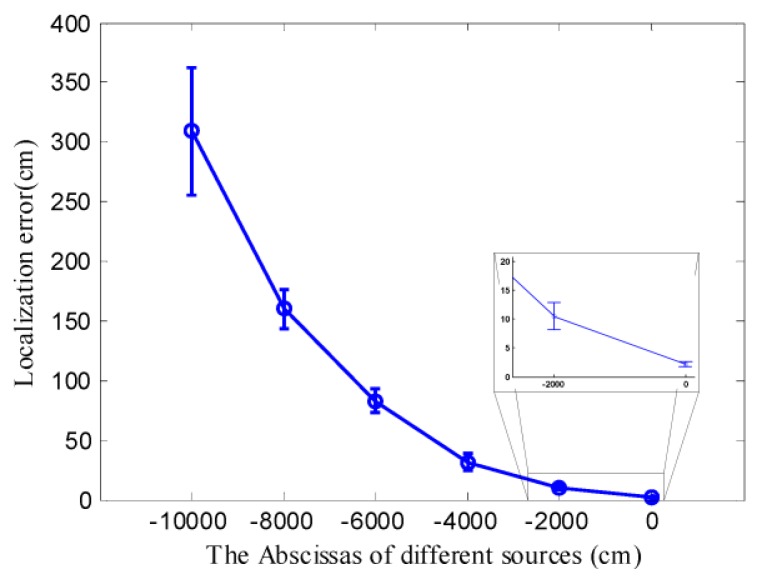
The statistical performance of locating a remote chemical source using DLSE. All the ordinates of these chemical sources were set as 4000 cm, thus, only the abscissas of the sources were displayed to denote the associated sources. The center and half-length of the error bar are the mean and standard deviation of the localization errors, respectively.

As shown in Figure 8d, DLSE succeeded in locating a remote chemical source which was located far away from the node deployment area. However, the results presented in Figure 8d was obtained based on limited times of simulation of locating the same source. To get a more convictive assessment of the performance of locating a remote chemical source, the actual chemical source was located at different locations far away from the node deployment area. For each location of the chemical source, 100 times of simulations were conducted, with the measurements collected by the nodes which were randomly re-deployed at the beginning of each simulation. TRR was not considered in these cases due to its poor convergence even when the actual chemical source was located in the node deployment area as shown in Figure 8a. Since the initial point can hardly influence the performance of DLSE, it was set as the centroid of the node deployment area, *i.e.*, (4000,4000) cm, in all of these simulations. The statistical results of these simulations were represented with error bars in Figure 9.

As shown in Figure 9, all of the chemical sources were successfully located with relatively small localization errors compared with the large sensor-source distances. The localization errors became larger with an increase in the distance between the source and the node deployment area. Because the theoretical concentration is inversely proportional to the sensor-source distance when other variables are kept constant according to Equation (11), the SNR of the measurements decreased, which would influence the localization performance, as the distance between the source and the node deployment area increased. However, the performance of any localization method based on concentration measurements might be influenced by the SNR of the measurements.

## 5. Real Experiment Results

In addition to the simulations, our methods were assessed in realistic experiments using a WSN which consists of twenty five real sensor nodes.

### 5.1. The Sensor Nodes and Realistic Environment

The sensor node was constructed based on the C51RF-CC2431 module (Wireless Dragon, Co. Ltd., Chengdu, China). Each of the nodes was equipped with a MiCS-5521 (SGX sensor Technology, Co. Ltd. Neuchatel, Switzerland) gas sensor to measure the chemical concentration. The nodes can communicate with the sink node via the wireless ZigBee protocol. The sensor nodes are able to reliably transmit the real-time sensing voltages of MiCS-5521 to the sink node. Due to the limitation of our currently available nodes, the wind speed was measured by two additional WindSonic anemometers (Gill, Co. Ltd., Hampshire, England).

**Figure 10.**
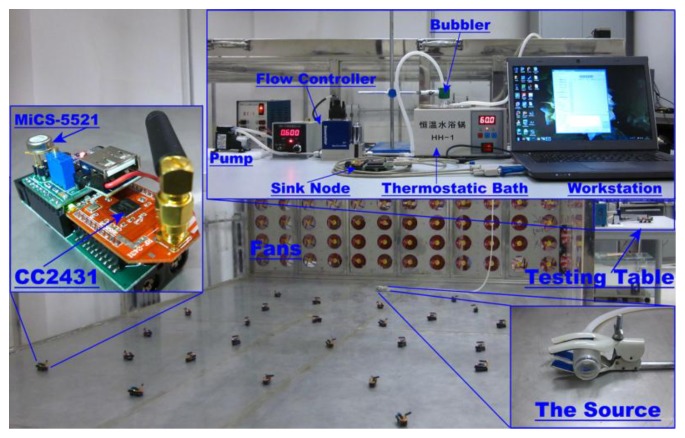
The general picture of the experimental installations. The bubbler was kept in a thermostatic bath so as to keep pressure inside the bubbler at a constant saturated vapor pressure. The pump and the flow controller were used to blow the saturated ethanol vapor out from the bubbler into the wind tunnel through a long PVC pipe.

The general picture of the experimental installations is shown in Figure 10. A reduced scale wind tunnel, which is a cuboid with 300 × 400 cm^2^ area by 90 cm height, was built up to create an approximately homogeneous wind field. The inlet and outlet of the wind tunnel are two parallel sides of the cuboid. An array of electrical fans was integrated in a flat rack, which was mounted in the wind inlet. Clean air was blown into the tunnel through the wind inlet. More details about the wind tunnel can be found in [25]. A pump and a flow controller were used to blow saturated ethanol vapor from the bubbler into the wind tunnel through a long PVC tube. The density of the saturated ethanol vapor in the bubbler was maintained by a thermostatic bath. The saturated vapor pressure and density can be calculated using Antoine's equation and the Clausius-Clapeyron's equation, respectively. Then, the product of the volume flow rate and the density of the saturated ethanol vapor can be considered as the theoretical release rate of the chemical source.

### 5.2. Sensor Calibration

**Figure 11.**
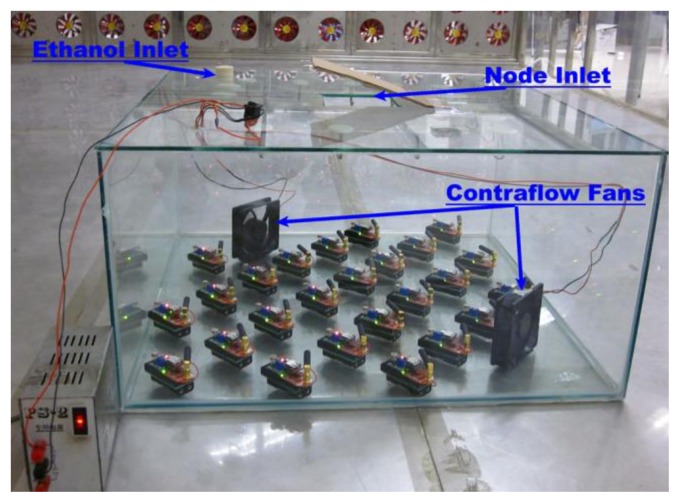
The experiment setup for calibrating the chemical sensors on the nodes. Different concentrations were created by dropping associated volumes of absolute ethanol into the glass box through the ethanol inlet. Both the ethanol inlet and the node inlet were sealed up during the calibration.

Before the sensor readings can be used to represent the concentration, the sensors should be calibrated to find an accurate mapping from the sensor readings to the concentration. Due to the controlled homogenous wind field, the concentration distribution and the sensor readings are approximately steady after a period of time [4]. Since this specific work condition approaches the closed sampling space [26], during the calibration process the sensor nodes were enclosed inside a closed glass box whose cubage is 108 L, as shown in Figure 11. The box was placed in the wind tunnel to make the sensors work under the same humidity and temperature like those in the process of localization. Then, multiple calibration points can be created by dropping in certain volumes of absolute ethyl ethanol and blowing them to accelerate their evaporation with convective wind. The relationship between the sensitivity *S*, *i.e.*, the ratio of the sensing resistance to the baseline resistance, and the concentration *C_s_* (in ppm) is well fitted to the transform relationship log *S* = *a* · log *C_s_* + *b*, in the range *C_s_* ∈ [10,1000] ppm [27]. Thus, the unknown parameters, *i.e.*, *a* and *b*, can be identified by fitting the steady sensing voltages and the associated actual concentrations to the calibration curve. Note that we refer to the final steady concentration, which was calculated by substituting the approximately steady voltage to the identified transform relationship, as the measurement in the rest of this section.

### 5.3. Identification of the Release Rate and the Eddy Diffusivity

**Figure 12.**
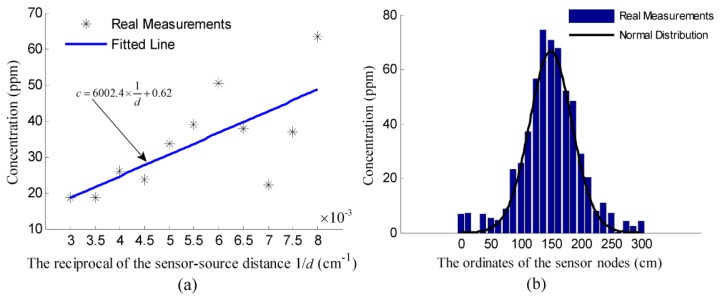
The experiment results of identifying *q* and *K*. (**a**) the measurements collected along the centerline of the plume were fitted to a line, of which the gradient can be considered as $\overset{\left(2\pi q_{0}\right)}{\;}/\underset{K}{\;}$. (**b**) the measurements along the line vertical to the plume centerline. The mean and standard deviation of these measurements were considered as the parameters for generating the normal distribution.

According to Equation (8), when *α* = −*π*, which means the sensor node lies on the positive abscissa axis of the Cartesian coordinate system in Figure 1b, the concentration of the node is:

(21) $$c=\frac{q}{2\pi Kd}=\frac{1}{2\pi }\chi \cdot \frac{1}{d}$$

where *χ* = *q*/*K* can be considered as a constant parameter during the process of localization. However, the premise is that the value of *q* and *K* are identified before the localization. Suppose a standard chemical source, which was releasing at a controlled release rate *q*_0_ = 0.01 g/s, was placed in the wind tunnel and taken as the origin of a Cartesian coordinate system like the one in Figure 1b. All the sensor nodes were placed along the positive abscissa axis. The abscissas of these nodes were set on the premise that the reciprocals of the distances from these nodes to the standard source should range from 0.003 to 0.008 in increments of 0.0005. The relationship of the concentration measurements of these nodes and the reciprocals of the associated distances was fitted into a straight line, which was shown in Figure 12a. Then, the value of $\overset{\chi_{0}}{\;}/\underset{2\pi }{\;},\chi_{0}=\overset{q_{0}}{\;}/\underset{K}{\;}$ can be identified as the gradient of the fitted line, and *K* can be calculated by dividing *q*_0_ by the identified value of *χ*_0_. According to Figure 12a, the function of the fitted line is *c* = 6002.4/*d* + 0.62, and the identified value of *K* is 103.15 cm^2^/s. Note that in real cases, the source to be located and the standard source may release simultaneously, so the concentration measurements used to identify *K* should be the increments of the measurements caused by the standard source.

With the identified value of *K*, we can further identify *q* by measuring the concentration along the centerline of the plume. The first step is to determine the unknown centerline of the plume caused by the source to be located. Because the distribution of the concentration measurements collected along the line perpendicular to the wind direction satisfies the normal law of errors [15], the unknown centerline can be determined as the line passing the maximum concentration point along the downwind direction. To verify this normal distribution, the twenty five sensor nodes were placed along a line vertical to the plume centerline. The measurements of these sensor nodes were plotted in Figure 12b. Since the measurements on the centerline of the plume caused by the source to be located satisfy $d=\overset{\chi }{\;}/\underset{\left(2\pi c\right)}{\;},\chi =\overset{q}{\;}/\underset{K}{\;}$, the value of $\overset{\chi }{\;}/\underset{2\pi }{\;}$ can be determined as the gradient of the line fitting the relationship between *d* and $\overset{1}{\;}/\underset{c}{\;}$. However, the distance between the sensor node and the unknown source, *i.e.*, *d*, was unknown. The gradient of the fitted line can only be approximately determined as $\overset{\Delta d}{\;}/\underset{\left(\Delta \overset{1}{\;}/\underset{c}{\;}\right)}{\;}$ when Δ*d* is relatively small. Thus, fifteen sensor nodes were placed with equal spacing Δ*d* = 20 cm on the centerline of the plume caused by the source to be located. The noisy concentration measurements of these sensor nodes, *i.e.*, *z_i_*, *i* ∈ [1, 15], and the associated value of Δ(1/*z*) = 1/*z_i_* − 1/*z_i_*_−1_,*i* ∈ [2,15] were shown in Table 1. In this experiment, the flow rate of the bubbler and the temperature of the bath were kept at 600 sccm (mL/min) and 60 °C, respectively. As mentioned in Section 5.1, the calculated theoretical release rate was 0.0078 g/s. As shown in Table 1, the mean of the values of Δ(1/*z*) = 1/*z_i_* − 1/*z_i_*_−1_ ,*i* ∈ [2,15] is 0.0037 ppm^−1^. Then, the value of *q* can be identified as 0.0071 g/s, which is close to the calculated theoretical release rate.

### 5.4. Experiment Results

**Figure 13.**
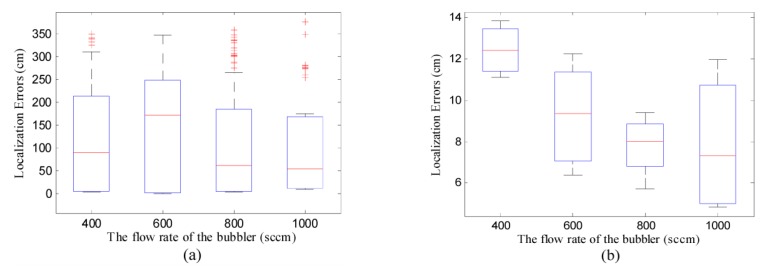
Boxplot of the localization errors in the four groups of tests. In both sub-figures, the maximum whisker lengths of the boxplots are set as 1. (**a**) The localization errors of TRR; (**b**) The localization errors of DLSE.

After the above-mentioned preliminaries, the performance of DLSE and TRR were assessed with real measurements. Note that, to avoid the complicated programming with the Zigbee protocol, the concentration measurements were transmitted to the workstation and the distributed implementation of DLSE was realized off-line. However, the online distributed implementation of DLSE is applicable after a further study on hardware design and embedded programming, which was not focused in this paper. Four scenarios were created by changing the flow rate of the bubbler from 400 sccm to 1000 sccm in increments of 200 sccm, while keeping the temperature of the thermostatic bath, the wind speed at 60 °C, 70 cm/s, respectively. The concentration threshold is set as 10 ppm to eliminate the measurements with low SNRs. Based on the measurements collected in each of the groups, one hundred of tests with different randomly selected initial points were conducted using TRR and DLSE. The boxplot of the localization errors in these groups were shown in Figure 13.

As shown in Figure 13a, although the minimum localization errors obtained using TRR were comparatively small, their overall distribution was highly fragmented and not symmetrical with respect to the median. Thus, the good performance of TRR is not guaranteed if the initial point is not properly chosen. In Figure 13b, DLSE succeeded in all of the tests with considerably high accuracy. The localization errors obtained based on the same group of measurements were generally symmetrically distributed with respect to the associated medians. In addition, higher flow rate of the bubbler, which means larger release rate of the chemical source and more participating scattered sensor nodes, results in smaller mean of localization errors.

## 6. Conclusions

In this paper, a distributed chemical source localization method is proposed for a WSN. Aiming to minimize the summands in the objective function of LSE-based CSL using WSNs, *i.e.*, the sum of squared measurement errors, individual local estimations of the source location are conducted on the participating scattered sensor nodes. This scheme avoids gathering the raw measurements and enables the realization of a distributed estimation method. The local estimates are convexly combined into the global estimate, which is used as the prior information for the local estimation at the next iteration of localization. The global estimate includes the information of all the local estimates in different degrees and enables collaboration among all the participating scattered sensor nodes. Our method shows a global convergence property with fast convergence rate, even when the actual chemical source is far away from the deployment area of the WSN. Thus, the initial point of our method can be preset as a random point before the process of measurement.

Our method iteratively incorporates the global information about the source location in a distributed manner so as to achieve a global convergence property. When the distribution models of radioactive substance and acoustic energy are utilized, our method can also be adapted to locate the radioactive and acoustic sources. Future works may cover deriving a theoretical probability of being the real chemical source for the local estimates. When such a probability is used as the weight of the associated local estimate, our method could evolve into a distributed particle filter.

## Figures and Tables

**Table 1.** The concentration measurements at the locations with equal spacing Δ*d* = 20 cm on the centerline of the plume caused by the source to be located.

**ID**	**1**	**2**	**3**	**4**	**5**	**6**	**7**	**8**	**9**	**10**	**11**	**12**	**13**	**14**	**15**
*z_i_*	54.89	49.58	41.03	33.48	35.16	31.15	27.68	21.52	26.53	22.67	19.39	19.03	17.11	11.91	15.17
Δ(1/*z*)*_i_*	NA	0.0019	0.0042	0.0055	0.0014	0.0036	0.0040	0.0103	0.0088	0.0064	0.0075	0.0009	0.0059	0.0255	0.0180
